# A Dual Mechanism of Cognition and Emotion in Processing Moral-Vertical Metaphors

**DOI:** 10.3389/fpsyg.2018.01554

**Published:** 2018-08-28

**Authors:** Dongxue Zhai, Yaling Guo, Zhongyi Lu

**Affiliations:** ^1^College of Education, Hebei Normal University, Shijiazhuang, China; ^2^School of Nursing, Chengdu Medical College, Chengdu, China; ^3^College of Foreign Languages, Hebei Normal University, Shijiazhuang, China; ^4^National Research Center of Foreign Language Education, Beijing Foreign Studies University, Beijing, China

**Keywords:** metaphor, moral concepts, vertical representations, morality, emotion

## Abstract

A moral concept involves two main factors: moral cognition (indicated by morality) and emotion (indicated by emotionality). The cognitive mechanism underlying moral metaphors on the vertical dimension (e.g., moral-up, immoral-down) was investigated in three experiments using implicit association tests. The results of Experiment 1 show a stronger association of “moral-up, immoral-down” between words high in morality and vertical space than between words low in morality and vertical space, which indicates that cognitive factors of morality facilitate the processing of vertical spatial metaphors of moral concepts. Experiment 2, employing moral words different in emotionality, reveals a stronger association of “moral-up, immoral-down” between words high in emotionality and vertical space than between words low in emotionality and vertical space, which shows that emotional factors of morality facilitate the processing of vertical spatial metaphors of moral concepts. A comparison between the two experiments suggests a faster response to emotion than to moral cognition and similar association strengths of the two factors with verticality. Using words high in morality and emotionality, Experiment 3 shows that a combination of the two conditions (i.e., high morality and high emotionality) leads to a stronger tie with verticality than either condition. The above three experiments indicate that both moral cognition and emotion facilitate the processing of vertical spatial metaphor of moral concepts, and the forces of the two, which jointly affect the metaphorical connection between morality and verticality, are basically equal, although the processing of emotionality is faster than that of morality.

## Introduction

The vertical spatial metaphor of moral concepts—and metaphors in general—have been studied from the embodiment perspective (e.g., Meier et al., 2007; Hill and Lapsley, 2009) since the publication of the two seminal works of Lakoff and Johnson (1980, 1999). According to the conceptual metaphor theory (Lakoff and Johnson, 1980), conceptual metaphorization occurs in a source domain consisting of concrete concepts we are familiar with (such as spatial orientations and physical entities) and a target domain comprising abstract concepts hard to understand or unfamiliar to us (such as time, morality). Metaphor is systematic mapping from a source domain to a target domain, projecting some characteristics of the former onto the latter (Lakoff, 1993). Thus, abstract concepts are understood and structured in terms of concrete concepts. Such cross-domain mappings are “at the heart of the unique human cognitive faculty of producing, transferring, and processing meaning” (Fauconnier, 1999, p. 1).

The embodied cognition theory (Lakoff and Johnson, 1999) regards most abstract concepts as metaphorical. Their source domains come from the body's sensorimotor system, and human beings usually perceive movement through space. Among spatial relationships, vertical (versus horizontal) experience is particularly salient, since verticality, the basic direction of human spatial perception, is a fundamental dimension in spatial expressions (Clark, 1973). While metaphors map a spatial position from source domains onto target domains, the image-schemas and their attendant logic are retained by the mappings (Lakoff and Turner, 1989, p. 99). One of such spatial metaphors is for morality, in which morality and immorality are conceptualized in terms of high or low position. The moral-spatial metaphors are significant to human cognition since they connect our basic vertical experience with our judgement about goodness or badness, which is necessary for our existence. The metaphor “MORALITY IS SPATIALITY,” therefore, has inspired a considerable amount of researches (Yu, 2016).

In line with the suggestion, most previous psychological studies on vertical spatial metaphor of morality have focused on its psychological reality and mapping, confirming “moral is up” and “immoral is down” (Meier et al., 2007; Wang and Lu, 2013) or just “moral is up” or “immoral is down” (Hill and Lapsley, 2009; Lawrence et al., 2011). Wang et al. (2016) found a strong implicit association between “moral is up” and “immoral is down” and a quicker response to “moral is up” in their Implicit Association Test (IAT) and ERP experiment. Lu et al. (2017) investigated the mapping of the vertical spatial metaphor of morality and reported an imbalanced, flexible bi-directional mapping between the two domains of morality and space.

In spite of the fruitful studies in this field, two questions remain to be answered: which of the three (i.e., cognitive factors or emotional factors or both) plays an important role in psychological reality and mapping of moral metaphors on the vertical dimension? If the answer is both, which of the two plays a more prominent role? This study attempts to explore these questions. Currently there are misconceived assumptions about moral and emotional valences. Moral stands for positive value while immoral negative, and thus moral words involve positive emotional valence while immoral words negative emotional valence. Consequently, moral value is regarded as identical to emotional value. In addition, the vertical spatial metaphor of moral concepts is closely related to that of emotional concepts and many previous studies have found a metaphorical connection between emotion and verticality, demonstrating that “positive is up and negative is down” (Wapner et al., 1957; Meier and Robinson, 2004; Wu et al., 2008; Henetz and Casasanto, 2009; Lü and Lu, 2013; Gottwald et al., 2015; Xie et al., 2015). Researchers also implicitly associate positive and negative ideas with left–right space (Casasanto, 2009; Casasanto and Chrysikou, 2011; de la Vega et al., 2012), and found a metaphorical connection between emotion and horizontality. These findings inspired our study of the dual mechanism.

There is a bi-directional but illogical inference between morality and emotion in past studies. What we try to clarify is that morality may denote positive emotion while immorality means negative, but it is not necessarily the case vice versa. Positively valenced words do not necessarily mean moral and negatively valenced words do not necessarily mean immoral. Previous studies on moral metaphors on the vertical dimension seemed to neglect the fact that moral concepts comprise morality and emotions. Therefore, only by investigating the two categories respectively in terms of their roles and their relationship can we explore the essence of vertical spatial representations of moral concepts.

In view of this, our study adopted the method of functional separation–that is, we controlled a component (such as morality) by making it constant–to observe the effects of changes in another component (such as emotionality). We separated morality from emotion by manipulating morality and emotionality of words so as to detect their respective mechanism. We systematically investigated the mechanisms of morality and emotion in processing moral vertical metaphors in three experiments. Experiment 1 focused on the mechanism of cognitive factors in vertical spatial metaphor of moral concepts; Experiment 2 explored the mechanism of emotional factors in vertical spatial metaphor of moral concepts; Experiment 3 examined the role of morality and emotionality in vertical spatial metaphor of moral concepts. A comprehensive analysis was conducted to compare their processing speed and strength, i.e., which is faster and which is stronger.

## Experiment 1

By employing the widely-used IAT, this experiment aims to find out whether differences exist in association strength between high vs. low morality and space when emotionality is kept constant. If both morality and emotionality influence the metaphorical tie between morality and verticality, there will be difference in the association strength with space between high and low morality when emotionality is controlled. To be specific, when emotionality remains the same, if words high in morality establish a stronger link with vertical space, the effect of morality in processing vertical metaphors of morality can be confirmed.

### Methods

#### Participants

Participants included a total of 36 randomly recruited undergraduates from a Chinese university in Hebei province (23 female, 13 male, Mean age = 19.9). All participants were Chinese native speakers and had normal or corrected-to-normal vision.

#### Materials

A large number of two-character Chinese words were selected from articles or books on morality and their degrees of morality and emotionality were evaluated. The procedures are as follows:

1. Establish a moral and immoral vocabulary. First, 269 two-character words concerning morality without explicit denotation of vertical space were selected into our Survey of Words' Moral Valence. Thirty participants randomly chosen were asked to assess the morality of each word by a 9-point rating of −4 to 4, (−4 = very immoral; 0 = no moral connotation; 4 = very moral); then we integrated 220 words out of the 269 words into our Survey of Words' Emotional Valence and asked 25 randomly selected participants to evaluate the emotional level of each word from −4 to 4 on a 9-point scale, (−4 = extremely negatively emotional, 0 = no emotional connotation, 4 = extremely positively emotional). After the assessment, the mean and standard deviation of morality and emotionality of all the words were calculated, and 179 words whose average values of morality and emotionality were both >1 and <-1 were selected to form the experimental material database of the present study. Word frequency information was obtained from “the Corpus System of Modern Chinese Research” of Beijing Language and Culture University (http://www.dwhyyjzx.com/cgi-bin/yuliao/).
2. Select words with high and low morality and words with high and low immorality. In Experiment 1, we selected 8 high morality words (morality *M* = 2.888, such as “诚信” which means “integrity,” “诚实” which means “honesty,” “宽容” which means “tolerance”) and 8 low morality words (morality *M* = 1.880, such as “纯朴” which means “unsophisticated,” “节约” which means “thrifty,” “勤勉” which means “diligence”) from the established material database. There was no significant difference in their emotional valence, word frequency or strokes (*t* = 1.268, *p* > 0.05; *t* = −0.316, *p* > 0.05; *t* = 0.535, *p* > 0.05), and they were only significantly different in morality, *t*_(1, 7)_ = 14.352, *p* < 0.001. We then selected 8 high immorality words (immorality *M* = −2.823, such as “诽谤” which means “slander,” “贿赂” which means “bribe,” “渎职” which means “malfeasance”) and 8 low immorality words (immorality *M* = −2.019, such as “刁钻” which means “tricky,” “自私” which means “selfish,” “粗暴” which means “rough”). The two types of words do not have significant difference in emotionality, frequency or strokes (*t* = −1.255, *p* > 0.05; *t* = −0.447, *p* = > 0.05; *t* = 0.282, *p* > 0.05), but have significant difference in immorality, *t*_(1, 7)_ = −8.882, *p* < 0.001 (see Appendix 1 in Supplementary Materials).

#### Procedure

Employing 2 (word types: moral words vs. immoral words) × 2 (morality: high vs. Low) × 2 (vertical positions: up vs. down) within-subject design, this experiment adopted an implicit association test (IAT; Greenwald et al., 1998), programmed with E-prime 2.0. The procedures were as follows:

1. Participants were first presented with “+” fixation point in the center of the screen for 500 ms, followed by moral words and immoral words randomly presented in the middle of the screen. Participants were required to categorize each word as moral or immoral quickly and accurately. They were instructed to press the “F” key if the word was moral and to press the “J” key if the word was immoral. The words remained on the screen until the participants responded.
2. The sign of asterisks “***” randomly appeared either on the upper side or the down side of the screen, signifying vertical spatial location, with the sign over the fixation point signaling up and under the fixation point signaling down. Participants were instructed to press the “F” key for up and “J” for down.
3. Moral words, immoral words, and vertical spatial words were presented together and participants were required to press the “F” key if the word was moral or up and to press the “J” key if the word was immoral or down. We labeled it Implicit Association Combined Task 1 (IAT1).
4. Participants were required to categorize words, but they were instructed to press the contrary key, that is, they press the “F” key if the word is immoral and press the “J” key if the word is moral.
5. Participants were presented with moral words, immoral words together with vertical spatial words and were required to press the “F” key if the word was immoral or up and press the “J” key if the word was moral or down. We labeled it as Implicit Association Combined Task 2 (IAT2). To avoid undesired sequence effects of combined tasks, half of them completed compatible task and then incompatible task, following the order of steps 1-2-3-4-5; half of them completed incompatible task and then compatible task, following the order of steps 4-2-5-1-3. Participants' reaction time (henceforth RT) were recorded as the dependent variable.

### Results

The accuracy rate of all participants of Experiment 1 was above 80%. We excluded trials with RT shorter than 300 ms and deleted 3 standard deviation below and above the mean (2.42% of trials). Inaccurate trials were not eliminated, according to Greenwald et al. (2003) who suggested that RT of inaccurate trials is meaningful and should be analyzed with that of accurate ones, for the RT of inaccurate trials is 500 ms longer than that of accurate ones and error rate tends to be higher in incompatible tasks.

Following the data-reduction procedure of Greenwald et al. (2003), we replaced RT of inaccurate trials with average reaction time plus 600 ms. The final data were analyzed using SPSS 17.0.

#### Analysis of reaction time

The transformed data according to the above criteria were analyzed, and the means of RT and standard deviation in compatible task (IAT1) and incompatible task (IAT2) were represented in Table 1.

**Table 1:** Mean reaction time and standard deviation of combined tasks (ms).

	***M***	***SD***
IAT1 (moral-up, immoral-down)	575	60
IAT2 (moral-down, immoral-up)	657	92

Paired samples *t*-tests were performed on mean RT, and the result indicated significant difference between mean RT of the two tasks, *t*_(1, 35)_ = −5.789, *p* < 0.001. Compared with RT in the incompatible task, participants' RT in compatible task was faster (the same key for moral and up; the same key for immoral and down), which indicated implicit association of “moral-up and immoral-down” between moral concepts and vertical spatial position.

A 2 (word type: moral vs. immoral) × 2 (task compatibility: compatible vs. incompatible) × 2 (morality: high vs. low) repeated measures ANONA was conducted and we found a main effect of word type, *F*_(1, 35)_ = 10.71, *p* < 0.01, η^2^ = 0.234, indicating that participants reacted to moral words (607 ms) significantly faster than immoral words (624 ms). There was a main effect of task compatibility, *F*_(1, 35)_ = 33.422, *p* < 0.001, η^2^ = 0.488, which means RT in compatible task (574 ms) is faster than incompatible task (657 ms). No morality main effect was found, *F*_(1, 35)_ = 0.268, *p* > 0.05. The interaction between morality and word type was not significant, *F*_(1, 35)_ = 2.844, *p* > 0.05. The interaction between morality and task compatibility was not significant, *F*_(1, 35)_ = 2.434, *p* > 0.05. The interaction between word type and task compatibility was not significant, *F*_(1, 35)_ = 0.366, *p* > 0.05. The interaction between morality, word type and task compatibility was not significant, *F*_(1, 35)_ = 0.858, *p* > 0.05.

#### Analysis of implicit association strength (D-value)

Greenwald et al. (2003) put forward a new algorithm for scoring the IAT, dividing the difference between two task means by the standard deviation of all the RT. This new algorithm, a RT-mean-variability calibrated measure, outperforms the conventional methods in avoidance of many drawbacks. The calculated value, *D*-value, stands for implicit association strength and reflects the effect size of IAT. The *D*-value of each participant was calculated and the mean was listed in Table 2.

**Table 2:** Mean of implicit association strength between moral concept and vertical space (D).

**Word type**	**Morality**	
	**High**	**Low**
Moral words	0.438	0.379
Immoral words	0.442	0.378

Paired samples *t*-tests were performed on the *D*-value and the results showed that the difference of *D*-value between high morality and low morality was significant, *t*_(1, 71)_ = 2.106, *p* < 0.05. The *D-*value of words high in morality (0.440) was significantly higher than that low in morality (0.378). The results indicate that words of high morality form a stronger tie with vertical space than words of low morality. Morality plays an important role in processing moral metaphors on the vertical dimension.

#### Analysis of error rate

In compatible task, there were 140 errors in the total 4,608 trials and the error rate was 3.04%, while in incompatible task, there were 242 errors and the error rate was 5.25%. An independent-sample test revealed significant difference in joint tasks with error rate in incompatible task significantly higher than in compatible task, *t*_(70)_ = −3.109, *p* < 0.05.

### Discussion

In Experiment 1, morality rated by participants signifies moral cognition. The higher morality is, the more moral the words are, and the more they can motivate moral concepts. The findings of RT are consistent with previous studies that have confirmed the metaphorical ties between morality and vertical space. However, repeated measures ANOVA of RT reported only the main effect of task compatibility and word type and no difference in RT between words high in morality and low morality. The results do not necessarily mean morality fails to work, for the analysis of D value indicates that association strengths between words high in morality and verticality were significantly stronger than that between low morality and verticality. According to Greenwald et al. (2003), as an improved scoring algorithm IAT, *D*-value can better reflect the potential association strengths between variables than RT and better reflect individual variance caused by association strengths other than irrelevant factors. The effects of experiment are thus better observed. We found significant difference in association strengths between words high vs. low in morality and vertical space and no significant difference in RT of words in high morality and low morality. Moral words high in morality activate participants' stronger morality and in turn stronger association strengths with vertical space, which demonstrates the facilitating effects of morality in processing vertical spatial metaphors of moral concepts.

## Experiment 2

The procedure was identical to Experiment 1 except that we controlled the morality of materials to detect whether there was difference in association strengths between vertical spatial concept and words with identical morality but different in emotionality. Presumably, if emotions work in processing vertical spatial metaphors of morality, words with high emotionality are more likely to establish stronger association with vertical space than words with low emotionality, when their morality is identical.

### Methods

#### Participants

Thirty-nine undergraduates from a Chinese university in Hebei province were recruited (22 female, 17 male, Mean age = 19.23). All participants were Chinese native speakers and had normal or corrected-to-normal vision.

#### Materials

We selected 16 moral words, 8 with high positive emotion (positive emotionality *M* = 2.96, such as “真诚” which means “sincere,” “贤良” which means “virtuous,” “忠诚” which means “loyal”) and 8 with low positive emotion (positive emotionality *M* = 2.34, such as “慷慨” which means “generous,” “无私” which means “selfless,” “和善” which means “kind”) from the established material database. There were no significant differences in morality, word frequency or strokes (*t* = 0.19, *p* > 0.05; *t* = −0.552, *p* > 0.05; *t* = −0.649, *p* > 0.05), but significant difference in positive emotionality, *t*_(1, 7)_ = 5.24, *p* < 0.01. We also selected 16 immoral words, 8 with high negative emotion (negative emotionality *M* = −2.98, such as “凶狠” which means “cruel,” “邪恶” which means “evil,” “摧毁” which means “destroy”) and 8 with low negative emotion (negative emotionality *M* = −2.435, such as “摧毁” which means “ridicule,” “刻薄” which means “mean,” “浪费” which means “waste”). The two types of words did not have significant difference in morality, frequency or strokes (*t* = −1.414, *p* > 0.05; *t* = −0.553, *p* > 0.05; *t* = 0.215, *p* > 0.05), but had significant difference in emotionality, *t*_(1, 7)_ = −4.85, *p* < 0.01 (see Appendix 2 for Experiment 2).

#### Experiment design and procedure

We performed a 2 (word types: moral words vs. immoral words) ×2 (emotionality: high vs. low) × 2 (vertical position: up vs. down) within-subject experiment whose procedure was identical to Experiment 1.

### Results

Among the 39 participants, three were removed because their accuracy rates were below 80% either in compatible combined task or incompatible combined task. We excluded trials with reaction time shorter than 300 ms and deleted 3 standard deviation below and above the mean (3.84% of trials). Error trials were not eliminated and analyzed as in Experiment 1, using SPSS 17.0. The mean of RT of each participant in IAT1and IAT2 and SD were calculated (see Table 3).

**Table 3:** Mean RT and SD of the two combined tasks (ms).

	***M***	***SD***
IAT1 (moral-up, immoral-down)	556	58
IAT2 (moral-down, immoral–up)	624	86

#### Analysis of reaction time

Paired samples *t*-tests were conducted on mean RT in the two combined tasks. The results revealed a significant difference in RT between IAT1 and IAT2, *t*_(1, 35)_ = −5.789, *p* < 0.001, indicating that participants' RT in compatible task (pressing the same key for moral and up, pressing another same key for immoral and down) was much shorter than incompatible task (pressing the same key for moral and down, pressing another same key for immoral and up). There was implicit association of “moral is up and immoral is down” between morality and verticality.

To detect the role of emotion in the metaphorical association, we performed a 2 (word types: moral words vs. immoral words) × 2 (task compatibility: compatible vs. incompatible) × 2 (emotionality: high vs. low) repeated measures ANOVA. There was a main effect of emotionality, *F*_(1, 35)_ = 38.893, *p* < 0.001, η^2^ = 0.526, indicating a faster RT of words high in emotionality (582 ms) than words low in emotionality (598 ms). There was a main effect of task compatibility, *F*_(1, 35)_ = 29.54, *p* < 0.001, η^2^ = 0.458, RT in compatible task (555 ms) was significantly shorter than that in incompatible task (625 ms). There was no word type main effect, *F*_(1, 35)_ = 2.211, *p* > 0.05. Additionally, the interaction effect between emotionality and word type was marginally significant, *F*_(1, 35)_ = 3.913, *p* = 0.056, η^2^ = 0.101. A further simple effect test revealed that RT of moral words high in emotionality was significantly shorter than words low in emotionality, *F*_(1, 35)_ = 4.98, *p* < 0.05. Similarly, RT of immoral words high in emotionality was significantly shorter than words low in emotionality, *F*_(1, 35)_ = 26.71, *p* < 0.001. There was no significant interaction effect between emotionality and task compatibility, *F*_(1, 35)_ = 1.994, *p* > 0.05. There was no significant interaction effect between word type and task compatibility, *F*_(1, 35)_ = 0.215, *p* > 0.05. There was no significant interaction effect was found between emotionality, word type and task compatibility, *F*_(1, 35)_ = 0.135, *p* > 0.05.

#### Analysis of implicit association strength (D-value)

The mean *D*-value was listed in Table 4 after the *D*-value of each individual was calculated.

**Table 4:** Mean of implicit association strength between moral words and vertical space (D).

**Moral word type**	**Emotionality**	
	**High**	**Low**
Moral words	0.368	0.293
Immoral words	0.396	0.329

Paired samples *t-*tests were performed on *D*-value and the results showed that the difference between high emotionality and low emotionality was significant, *t*_(1, 69)_ = 2.142, *p* < 0.05, indicating a stronger association (0.382) of space and words high in emotionality than words low in emotionality (0.311). The results of Experiment 2 suggested that implicit metaphorical links between words high in emotionality and vertical space were stronger than between words low in emotionality and vertical space. Emotions play a role in processing moral metaphors in vertical space.

#### Analysis of error rate

In compatible task, there were 175 errors in the total 4,608 trials and the error rate was 3.80%, while in incompatible task, there were 290 errors and the error rate was 6.29%. An independent-sample test revealed significant difference in joint tasks with error rate in incompatible task significantly higher than in compatible task, *t*_(70)_ = −3.145, *p* > 0.05.

### A comparative analysis of results of experiment 1 and experiment 2

Both results of Experiments 1 and 2 suggest that morality and emotion play their respective role in processing vertical spatial metaphor of moral words. We made a comparative analysis of RT and association strengths of the two experiments to investigate the speed and strength of morality and emotionality, trying to know which was faster and stronger (see Table 5).

**Table 5:** Mean RT of experiment 1 and experiment 2 (ms) and *D*-value.

**Word type**	**Morality**				**Emotionality**			
	**High**		**Low**		**High**		**Low**	
	**RT**	**D**	**RT**	**D**	**RT**	**D**	**RT**	**D**
Moral words	603	0.438	612	0.379	582	0.368	591	0.293
Immoral	627	0.442	622	0.378	583	0.396	606	0.329

We conducted a 2 (factor type: high morality vs. high emotionality) × 2 (word type: moral vs. immoral) double factor variance analysis, using RT of words high in morality and emotionality as dependent variable. A factor type main effect was found, *F*_(1, 70)_ = 9.567, *p* < 0.01, η^2^ = 0.033, the RT of words high in emotionality (582 ms) was much shorter than RT of words high in morality (615 ms). There was no main effect of word type, *F*_(1, 70)_ = 1.304, *p* > 0.05, and no interaction effect was observed between factor type and word type, *F*_(1, 70)_ = 1.085, *p* > 0.05. Using RT of words low in morality and emotionality as dependent variable, we performed a 2 (factor type: low morality vs. low emotionality) × 2 (word type: moral vs. immoral) double factor variance analysis. No factor type main effect was found, *F*_(1, 70)_ = 3.069, *p* > 0.05, which suggested that there was no significant difference between RT of words low in morality and low in emotionality. There was no significant main effect of word type, *F*_(1, 70)_ = 1.403, *p* > 0.05. There was no significant interaction effect between factor type and word type, *F*_(1, 70)_ = 0.049, *p* > 0.05. The above analysis revealed that the processing speed of words high in emotionality was significantly faster than words high in morality. Words low in emotionality did not differ significantly from words low in morality, which means words more emotionally salient were processed more quickly, that is, emotional factors of moral concept were processed faster than moral factors.

Using the *D*-value of words high in morality in Experiment 1 and words high in emotionality in Experiment 2 as dependent variables, we performed a 2 (factor type: high morality vs. high emotionality) × 2 (word type: moral vs. immoral) double factor variance analysis and the result showed no main effects or interaction effects. Using the *D*-value of words low in morality in Experiment 1 and words low in emotionality in Experiment 2 as dependent variables, we performed a 2 (factor type: low morality vs. low emotionality) × 2 (word type: moral vs. immoral) double factor variance analysis and the result showed no main effects or interaction effects. A comparative analysis of association strength indicated that morality and emotionality both exert equally powerful influence in processing moral metaphors on the vertical dimension.

### Discussion

A comparative analysis of RT in of IAT1 and IAT2 in Experiment 2 confirmed the psychological reality of the metaphor “moral is up and immoral is down.” The 3 factor (emotionality, word type, task compatibility) repeated measures ANOVA reported a main effect of emotionality. Participants' reaction was significantly quicker to words high in emotionality than to words low in emotionality. But in Experiment 1, no significant difference was found in RT of words different in morality. What accounts for the variance?

It is partly due to the fact that in moral recognition or decision, the processing of emotions is automatic and fast since emotions are fundamental and intuitive. On the contrary, morality requires rational reflection and inference, therefore the process is controllable and calm (Maiese, 2014). Our conclusion was consistent with the finding of Kousta et al. (2011) that abstract concepts more emotionally loaded were faster processed.

We compared RT of two experiments and found RT of words high in emotionality in Experiment 2 was significantly shorter than that of words high in morality in Experiment 1, which indicated that emotional salience did affect processing speed. In addition, we compared the RT of words with different factor type in Experiment 1 and Experiment 2 and observed a faster processing speed of words high in emotionality. But a comparative analysis found no significant difference in the *D*-value between high morality and high emotionality and no significant difference in the *D*-value between low morality and low emotionality. The findings suggest a faster speed of emotion in processing vertical spatial metaphor of moral concepts but equal association strengths with morality.

## Experiment 3

We employed words high in morality and emotionality to investigate the effects of morality and emotion in processing vertical spatial metaphors of moral concepts. Presumably, if the two are correlated with each other and contribute to processing of moral-vertical metaphors, words high in morality and emotionality will be most strongly associated with verticality and show the highest *D*-value in IAT.

### Methods

#### Participants

Participants included a total of 38 randomly recruited undergraduates from a Chinese university in Hebei province (22 female, 16 male, Mean age = 20.47). All participants were Chinese native speakers and had normal or corrected-to-normal vision, having no difficulty in reading.

#### Materials

We selected moral words with high morality and high positive emotionality and selected immoral words with high immorality and high negative emotionality. We selected 8 moral words and 8 immoral words from the vocabulary. The mean value of morality and emotionality was above 2.8 (moral words: morality *M* = 2.875, positive emotionality *M* = 2.998; immoral words: immorality *M* = −2.943, negative emotionality *M* = −2.990). Words like “慈善” (charity), “美德” (virtue), and “仁德” (benevolence) belonged to the category of high morality with high positive emotionality, while words like “罪恶” (sin), “欺骗” (deceive), and “污辱” (insult) were rated as highly immoral and highly negative in emotion. The 16 words did not have significant difference in frequency or strokes (*t* = 0.928, *p* > 0.05; *t* = −0.689, *p* > 0.05), but had significant difference in morality, *t*_(1, 7)_ = 179.915, *p* < 0.001 and in emotionality, *t*_(1, 7)_ = 42.506, *p* < 0.001 (see Appendix 3 for Experiment 3).

#### Experiment design and procedure

We performed a 2 (word types: moral words vs. immoral words) × 2 (vertical position: high vs. low) within-subject experiment whose procedure is identical to Experiment 1 and 2.

### Results

Among the 38 participants, two were removed because their accuracy rate was below 80%. We excluded trials with RT shorter than 300 ms and deleted 3 standard deviation below and above the mean (2.47% of trials). Error trials were not eliminated and analyzed as in Experiments 1 and 2, using SPSS 17.0.

#### Analysis of reaction time

We calculated Mean RT and SD of each participant in compatible task IAT1 and incompatible combined task IAT2 (see Table 6).

**Table 6:** Mean RT and SD of IAT1 & IAT2 (ms).

	***M***	***SD***
IAT1 (moral-up, immoral-down)	582	84
IAT2 (moral-down, immoral-up)	708	113

Paired samples *t-*tests of mean RT in two tasks indicated that there was significant difference in mean RT between IAT1 and IAT2, *t*_(1, 35)_ = −6.369, *p* < 0.001. Participants' RT in IAT1 (moral-up, immoral-down) was significantly shorter than RT in IAT2 (moral-down, immoral-up). The results suggested that metaphorical associations exist between moral words and verticality, showing a tendency of moral words linking to up and immoral words to down.

#### Implicit association strength analysis (*D*-value)

The *D*-value of each participant was calculated and the mean *D-*value of moral words and immoral words was represented in Table 7.

**Table 7:** Mean association strength of morality and verticality (D).

	**Word type**	
	**Moral words**	**Immoral words**
Association strength	0.630	0.535

Paired samples *t-*tests of the *D*-value of moral words and immoral words reported no significant difference in the *D-*value, *t*_(1, 35)_ = 1.856, *p* > 0.05, which means moral words and immoral words have similar association strength with vertical positions.

#### Analysis of error rate

In compatible task, there were 73 errors in the total 2,304 trials and the error rate was 3.02%, while in incompatible task, there were 142 errors and the error rate was 5.11%. An independent-sample test revealed a significant difference in joint tasks with error rate in incompatible task significantly higher than in compatible task, *t*_(70)_ = −3.028, *p* < 0.05.

## A comprehensive analysis of IAT effects

### A comprehensive variance analysis of reaction time of different word types in three experiments

We explored vertical spatial metaphor through words different in emotionality (high vs. low) and words high in morality and emotionality in three experiments. Besides, we performed a 3 (factor type: high morality in Experiment 1, high emotionality in Experiment 2, high morality and emotionality in Experiment 3) × 2 (word type: moral vs. immoral) double factor variance analysis and found no main effect of word type, *F*_(2, 105)_ = 1.880, *p* > 0.05, no significant interaction effect between factor type and word type, *F*_(2, 105)_ = 0.447, *p* > 0.05, but a main effect of factor type, *F*_(2, 105)_ = 13.879, *p* < 0.001, η^2^ = 0.061. Multiple comparisons indicated that:

1. There was a significant difference in RT between words high in morality in Experiment 1 and words high in emotionality in Experiment 2 (*p* < 0.01), to be specific, RT of high morality (615 ms) was significantly longer than high emotionality (582 ms).
2. The difference in RT was significant between words high in morality in Experiment 1 and high in morality and emotionality in Experiment 3 (*p* < 0.05); RT of high morality (615 ms) was significantly shorter than high morality and emotionality (645 ms).
3. There was a significant difference in RT between words high in emotionality in Experiment 2 and words high in morality and emotionality in Experiment 3 (*p* < 0.001); RT of words high in emotionality (582 ms) was significantly shorter than words high in morality and emotionality (645 ms).

Somewhat surprisingly, RT of words high in emotionality in Experiment 2 was shortest; RT of words high in morality in Experiment 1 was longer; RT of words high in morality and emotionality in Experiment 3 was longest. As have been discussed in Experiment 2, emotions were processed faster, while morality was processed more slowly for its involvement of reflection and inference. Accordingly, words high in emotionality were processed faster than words high in morality. Words high in morality and emotionality were processed most slowly, which may be caused by mutual effect of cognition and emotion, with one undermining the other.

### A comprehensive variance analysis of association strength of different word types in three experiments

The above analysis suggested that both morality and emotion work in processing moral metaphors on the vertical dimension, and their roles are similar. Then it is necessary to examine whether cognition and emotion work separately or together. We performed a 3 (factor type: high morality in Experiment 1, high emotionality in Experiment 2, high morality and emotionality in Experiment 3) × 2 (word type: moral vs. immoral) double factor variance analysis, using the *D*-value of words high in morality in Experiment 1, words high in emotionality in Experiment 2 and words high in morality and emotionality in Experiment 3 as dependent variables within subjects. We found no main effect of word type, *F*_(2, 105)_ = 0.108, *p* > 0.05, no significant interaction effect between factor type and word type *F*_(2, 105)_ = 0.363, *p* > 0.05, but a main effect of factor type, *F*_(2, 105)_ = 3.652, *p* < 0.05, η^2^ = 0.034. Multiple comparisons indicated that:

1. No significant difference in the *D*-value existed between words high in morality in Experiment 1 and words high in emotionality in Experiment 2 (*p* > 0.05).
2. The difference in the *D*-value was significant between words high in morality in Experiment 1 and high in morality and emotionality in Experiment 3 (*p* = 0.064); the *D*-value of words high in morality and emotionality was significantly larger than words high in morality.
3. There was a significant difference in the *D*-value between words high in emotionality in Experiment 2 and words high in morality and emotionality in Experiment 3 (*p* < 0.01); the *D*-value of words high in morality and emotionality was significantly larger than words high in emotionality.

Words high in morality and emotionality in Experiment 3 form a significantly stronger association with verticality than words in Experiments 1 and 2, which means that words with two high valences form a stronger tie with verticality than words with one high valence. Morality and emotion can work in processing moral metaphor on the vertical dimension, respectively and jointly.

## General discussion

This study investigated mechanism of moral metaphors on the vertical dimension by evaluating morality, emotionality and their mutual effects through three experiments employing IAT.

In Experiment 1, using high and low morality as dependent variables, we controlled emotionality at the moderate level and found words high in morality establish a stronger association strength with vertical space than words low in morality. That is, high morality invokes stronger implicit metaphorical association of “moral-up and immoral-down.” A conclusion can be drawn that morality plays an indispensable role in processing moral concepts in vertical spatial position by contributing to vertical representation of moral concepts.

In Experiment 2, we controlled morality at the moderate level and distinguished high and low emotionality and observed a quicker response to words high in emotionality. It suggested that higher emotion activates faster processing of moral metaphors in verticality. The D measure also demonstrated that words high in emotionality have stronger association with verticality than words low in emotionality. Results of RT and *D*-value both indicated emotions, as morality does, facilitate processing of moral metaphors in verticality.

But which works faster and more powerfully? To answer the question, we made a comparison of RT and *D*-value of words with different factor types in Experiments 1 and 2 and found a quicker response to high emotion than any other condition, and no significant difference in *D*-value between high morality and high emotionality, low morality and low emotionality. In vertical representations of moral concepts, emotion is processed faster than morality and the two factors demonstrate similar association strength with verticality.

While Experiments 1 and 2 investigated morality and emotion respectively, Experiment 3 focused on words high in morality and emotionality, exploring their mutual effect in processing moral metaphors on the vertical dimension. The comprehensive variance analysis of different factor types in the three experiments revealed that high morality, along with high emotionality, had stronger association with verticality than high emotionality or high morality, indicating a correlation and a mutual effect of morality and emotion.

## Conclusion

1. Moral concepts are represented on the vertical dimension in the way “moral-up and immoral-down” and morality and emotion play critical roles in the process.
2. Both morality and emotion facilitate processing of moral metaphors in verticality, exerting similar force on implicit association. However, emotion is processed more quickly than morality.
3. When both moral and emotional factors in morality are salient, the association strength between verticality and morality will be highest, which can be interpreted as a mutual effect of cognition and emotion in processing moral metaphors in verticality.

## Limitations and future directions

Limitations of the study lie in the fact that all the participants were undergraduates and those from different educational background or at different age were not investigated. The characteristics of this sample may affect the results and introduce bias, making it difficult to generalize for the entire community. Future research may focus on participants of other educational background, from different regions of the world and at different age.

## Ethics statement

This study was carried out in accordance with the recommendations of the Committee for Protecting Human and Animal Subjects, Hebei Normal University. Parents gave written informed consent in accordance with the Declaration of Helsinki. The protocol was evaluated and approved by the Human and Animal Protection Committee of Hebei Normal University.

## Author contributions

DZ was involved in study design, data acquisition, data analysis, and manuscript writing. YG was involved in manuscript writing and revision. ZL was involved in the study design, the development of measures, and commented on the manuscript.

### Conflict of interest statement

The authors declare that the research was conducted in the absence of any commercial or financial relationships that could be construed as a potential conflict of interest.
