# Supplementary material for: A Dual Mechanism of Cognition and Emotion in Processing Moral-Vertical Metaphors
Source: Front Psychol. 2018 Aug 28;9:1554. doi: 10.3389/fpsyg.2018.01554 (PMC6122286; doi:10.3389/fpsyg.2018.01554)
Supplement: Supplementary file 1 [file Data_Sheet_1.pdf]

Appendix 1

Experiment 1

(1) Moral words with high morality

Words	morality (Independent variable)	Emotionality (controlled variable)	frequency (controlled variable)	strokes (controlled variable)
诚信<br>integrity	3.3	2.72	1.63	17
守信<br>trustworthy	2.9	2.52	0.81	15
宽容<br>tolerant	2.9	2.6	8.15	20
正直<br>upright	2.73	2.68	8.96	13
信义<br>loyalty	2.93	2.36	1.63	12
诚实<br>honest	3.00	2.6	13.85	16
信用<br>credit	2.67	2.2	8.96	14
仁慈<br>merciful	2.67	2.76	4.07	17
mean	2.888	2.555	6.01	15.50

(2) Moral words with low morality

Words	morality (Independent variable)	Emotionality (controlled variable)	frequency (controlled variable)	strokes (controlled variable)
体谅<br>considerate	1.97	2.44	15.48	17
贤惠<br>amiable	2.2	2.6	2.44	20
质朴<br>guileless	1.97	2.28	4.07	14
节约<br>thrift	1.9	2.32	20.37	10
勤勉<br>diligence	1.87	2.4	0.81	22
纯朴<br>unsophisticated	1.87	2.44	5.70	12
亲和<br>affinity	1.73	2.52	0.81	17
豪迈<br>heroic	1.53	2.6	5.70	20
mean	1.88	2.45	6.92	16.50

(3) Immoral words with high immorality

Words	morality (Independent variable)	Emotionality (controlled variable)	frequency (controlled variable)	strokes (controlled variable)
贿赂<br>bribe	-3.17	-2.36	4.07	20
诽谤<br>slander	-2.8	-2.76	0.81	22
渎职<br>malfeasance	-2.67	-2.6	1.63	22
伪善<br>insincerity	-2.97	-2.72	0.81	18
虚伪<br>hypocritical	-2.8	-2.76	13.04	17
残酷<br>merciless	-2.5	-2.76	15.48	23
丑恶<br>repulsive	-2.57	-2.6	7.33	14

(4) Immoral words with low immorality

Words	morality (Independent variable)	Emotionality (controlled variable)	frequency (controlled variable)	strokes (controlled variable)
自私<br>selfish	-2.07	-2.52	8.15	13
粗暴<br>rough	-2.37	-2.72	6.52	26
刁钻<br>tricky	-1.67	-2.4	2.44	12
腐朽<br>decay	-1.97	-2.76	8.96	21
虚假<br>sham	-2.3	-2.68	12.22	22
狂妄<br>arrogant	-1.9	-2.68	2.44	13
粗鄙<br>vulgar	-1.7	-2.6	1.63	24

贪婪<br>greedy	-3.1	-2.76	3.26	19	欺负<br>bully	-2.17	-2.44	13.85	18
mean	-2.823	-2.665	5.81	19.38	mean	-2.019	-2.600	7.03	18.63

Appendix 2

**Experiment 2**									
(1) Moral words high in positive emotionality					(2) Moral words low in positive emotionality				
Words	morality<br>(controlled variable)	Emotionality<br>(Independent variable)	frequency<br>(controlled variable)	strokes<br>(controlled variable)	Words	morality<br>(controlled variable)	Emotionality<br>(Independent variable)	frequency<br>(controlled variable)	strokes<br>(controlled variable)
真诚<br>sincere	2.67	3.08	39.11	18	无私<br>Selfless	2.57	2.56	34.22	11
贤良<br>virtuous	2.5	3.04	0.81	15	和善<br>kind	2.3	2.56	1.63	20
慈爱<br>loving	2.53	2.96	0.81	23	慷慨<br>generous	2.33	2.52	7.33	26
厚道<br>kindness	2.3	2.84	0.81	21	和睦<br>harmony	2.33	2.52	20.37	21
爱心<br>love	2.53	2.92	13.85	14	感恩<br>gratitude	2.5	2.48	1.63	23
忠实<br>loyal	2.4	3	7.33	16	宽恕<br>forgive	2.33	1.64	2.44	20
宽厚<br>lenience	2.3	2.88	2.44	19	仁厚<br>clemency	2.4	2.32	3.26	13
真挚<br>cordial	2.23	2.96	4.89	20	信誉<br>reputation	2.6	2.12	14.67	22
mean	2.433	2.96	8.76	18.25	mean	2.42	2.34	10.69	19.50
(3) Immoral words high in negative emotionality					(4) Immoral words low in negative emotionality				
Words	morality<br>(controlled variable)	Emotionality<br>(Independent variable)	frequency<br>(controlled variable)	strokes<br>(controlled variable)	Words	morality<br>(controlled variable)	Emotionality<br>(Independent variable)	frequency<br>(controlled variable)	strokes<br>(controlled variable)
凶狠<br>cruel	-2.5	-2.96	4.89	13	亵渎<br>profane	-2.7	-2.72	0.81	23
摧残<br>destroy	-2.73	-3	5.70	22	讥笑<br>ridicule	-2.33	-2.6	1.63	14
狰狞<br>ferocious	-2.07	-2.76	4.07	17	狡猾<br>cunning	-2.47	-2.56	5.70	21
禽畜<br>beasts	-2.53	-2.96	0.81	22	刻薄<br>mean	-2.43	-2.52	3.26	24
践踏<br>trample	-2.53	-2.8	2.44	26	徇私<br>favoritism	-2.47	-2.32	0.81	16

败坏 corrupted	-2.6	-2.88	2.44	12	无情 ruthless	-2.23	-2.32	16.29	15
邪恶 evil	-2.77	-3.36	5.70	16	违约 default	-2.2	-2.28	1.63	13
兽欲 wild desire	-2.77	-3.12	0.81	22	浪费 waste	-2.3	-2.16	6.52	20
mean	-2.563	-2.98	3.36	18.75	mean	-2.391	-2.435	4.58	18.25

Appendix 3

**Experiment 3**									
(1) Moral words high in morality and positive emotionality					(2) Immoral words high in immorality and negative emotionality				
Words	morality	Emotionality	frequency	strokes	Words	morality	Emotionality	frequency	strokes
	(Independent variable)	(Independent variable)	(controlled variable)	(controlled variable)		(Independent variable)	(Independent variable)	(controlled variable)	(controlled variable)
仁爱 philanthropy	2.93	3.2	0.81	14	阴险 nefarious	-2.87	-3.24	0.81	15
慈善 charity	2.93	2.72	7.33	25	罪恶 sin and crime	-2.90	-3	6.52	23
孝道 Filial piety	2.90	2.92	2.44	18	欺骗 deceive	-2.93	-2.92	20.37	24
忠孝 loyal and filial	3.10	2.88	0.81	14	污辱 insult	-2.93	-3.32	3.26	16
仁德 benevolence	2.80	3.16	0.81	19	掠夺 plunder	-2.97	-2.92	2.44	17
公正 fair	2.80	3.08	24.45	9	卑劣 despicable	-2.97	-2.92	1.63	14
善良 goodness	2.77	3.32	38.29	19	侮蔑 scorn	-2.97	-3.08	9.78	23
美德 virtue	2.77	2.7	18.74	23	阴谋 conspiracy	-3.00	-2.52	9.78	17
mean	2.875	2.998	11.71	17.63	mean	-2.943	-2.99	6.83	18.63
